# Supplementary material for: RNF19A-mediated ubiquitination of BARD1 prevents BRCA1/BARD1-dependent homologous recombination
Source: Nat Commun. 2021 Nov 17;12:6653. doi: 10.1038/s41467-021-27048-3 (PMC8599684; doi:10.1038/s41467-021-27048-3)
Supplement: Supplementary file 1 — Supplementary Information [file 41467_2021_27048_MOESM1_ESM.pdf]

RNF19A-mediated Ubiquitination of BARD1 Prevents BRCA1/BARD1 Dependent Homologous Recombination

Supplementary Information (Supplementary Figures 1-7 and Tables 1-2)

Zhu et al.

Supplementary Figure1

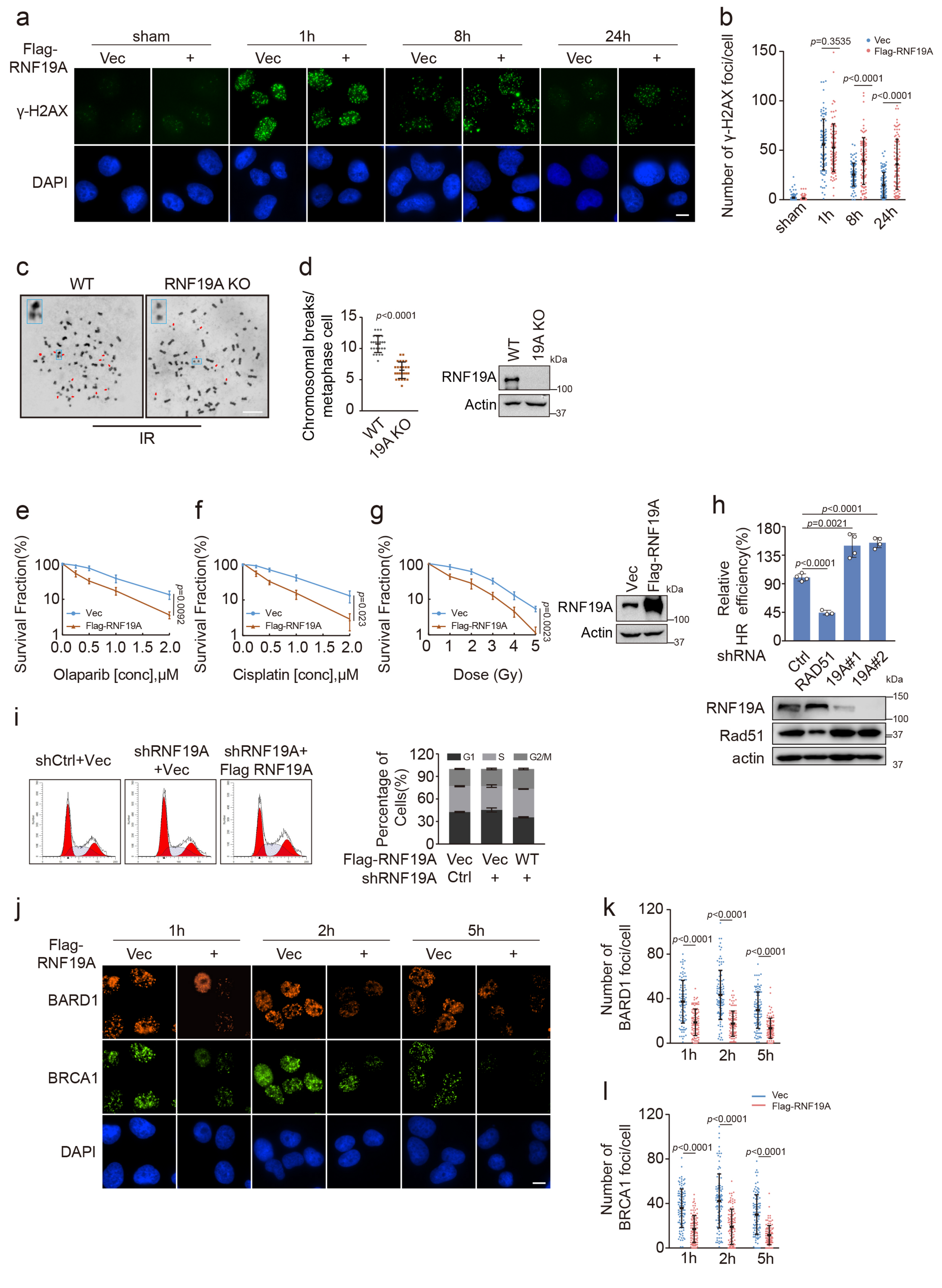

Supplementary Fig.1 RNF19A regulates DNA damage repair. a-b U2OS cells stably expressing Vec or Flag-RNF19A were treated with or without IR (4 Gy, 1 h). γ-H2AX foci before or 1/8/24 h after IR were detected by immunofluorescence (**a**). Nuclei were visualized with DAPI (blue). Quantification of focus signals per cell (n=100) is shown in (**b**). Scale bar, 10μM. **c-d** Representative images of metaphases prepared from IR-treated (0.5Gy), wild-type (WT) and RNF19A knockout (KO) U2OS cells. Arrows indicate the chromosomal breaks (**c,** magnification 1,000X). The blue boxes are enlarged representative breaks. Quantification of chromosomal breaks per cell (n=25) is shown in (**d**). Scale bar, 10μM. **e-g** Sensitivity of U2OS cells stably expressing Vec or Flag-RNF19A to Olaparib (**e**), cisplatin (**f**) and IR (**g**) was assessed using colony formation assay. **h** HR efficiency of control (Ctrl) and RNF19A knockdown HEK293T cells were assessed using the DR-GFP reporter assay. RAD51 knockdown cells were used as a positive control. Error bars represent means ± s.d. of four independent experiments. **i** Cell cycle was analyzed by flow cytometry in control (Ctrl) and RNF19A knockdown MDA-MB-231 cells stably expressing Vec or Flag-RNF19A. Error bars represent means ± s.d. of three independent experiments. **j-l** U2OS cells stably expressing Vec or Flag-RNF19A were treated with IR (4 Gy). BARD1 and BRCA1 foci 1/2/5 h after IR were detected by immunofluorescence (**j**). Quantification of focus signals per cell (n=100) is shown in (**k** and **l**). Scale bar, 10μM. Error bars represent means ± s.d. of three (**b, d-g, i, k** and **l**) or four (**h**) independent experiments. *p* values are determined by unpaired two-sided t-test in **b, d-i, k** and **l**.

Source data are provided as a Source Data file.

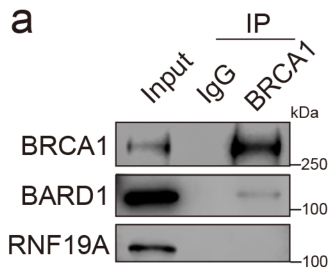

Supplementary Fig.2 RNF19A interacts with BARD1. a HEK293T cell lysates were subjected to immunoprecipitation with control IgG or BRCA1 antibodies and immunoblotted with the indicated antibodies.

Source data are provided as a Source Data file.

Supplementary Figure3

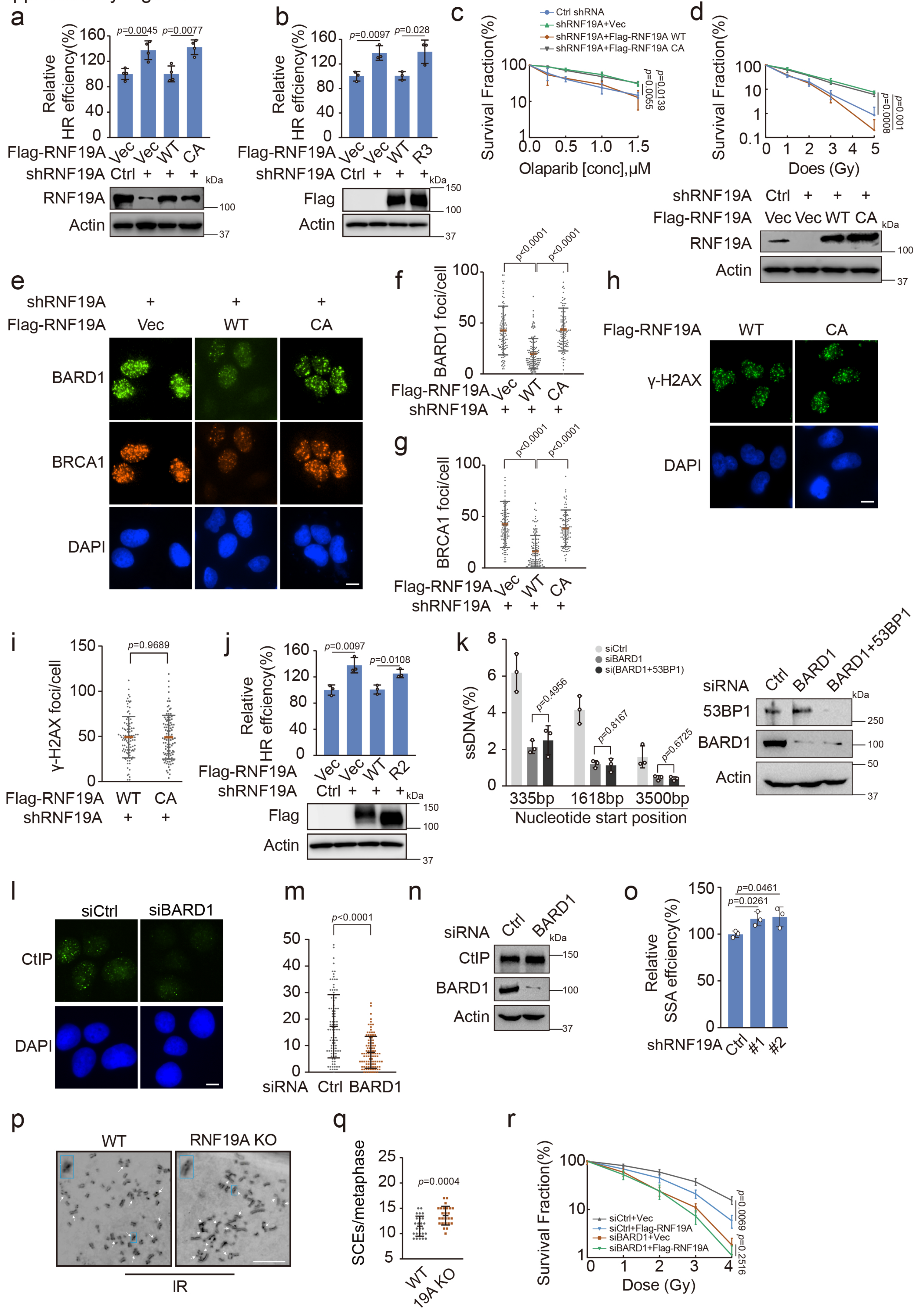

Supplementary Fig.3 The ubiquitinating enzyme activity of RNF19A is essential for its functions in HR. a-b, j Control (Ctrl) and RNF19A knockdown HEK293T cells stably expressing indicated plasmids were subjected to DR-GFP based HR assay. **c-d** Control (Ctrl) and RNF19A knockdown U2OS cells stably expressing indicated plasmids were subjected to colony formation assay for assessment of the sensitivity to Olaparib (**c**) and IR (**d**). **e-g** RNF19A knockdown U2OS cells stably expressing indicated plasmids were treated with IR (2 Gy, 1 h). BARD1 and BRCA1 foci were detected by immunofluorescence(**e**). Quantification of focus signals per cell (n=105) is shown in (**f**): BARD1 and (**g**): BRCA1. Scale bars, 10μM. **h-i** RNF19A knockdown U2OS cells stably expressing WT or C316A(CA) Flag-RNF19A were treated with IR (2 Gy, 1 h). γ- H2AX foci were detected by immunofluorescence (**h**). Quantification of focus signals per cell (n=100) is shown in (**i**). Scale bars, 10μM. **k** U2OS ER-*Asi*SI cells transfected with control, BARD1 or/and 53BP1 siRNAs for 4 8 h and were pretreated with 300 nM 4-OHT for 4 h before digest and measurement of DNA resection. **l-n** U2OS cells were transfected with control or BARD1 siRNAs for 48 h and treated with IR (5 Gy, 3 h). CtIP foci were detected by immunofluorescence (**l**) and quantified by focus signals per cell (**m**, n=100). The protein level was detected by Western blotting (**n**). scale bars, 10μM. **p-q** WT or RNF19A KO U2OS cells were labelled with 20 μM BrDU around two cell cycles after IR (0.5 Gy) treatment. (**p**) Representative metaphase spreads showing SCEs, magnification 1,000X. Arrows indicate visible SCEs, the blue boxes are enlarged representative SCEs. (**q**) Quantification of SCEs per cell (n=25). Scale bar, 10μM. **r** Indicated U2OS cells were subjected to colony formation assay for assessment of response to IR. Error bars represent means ± s.d. of three (**b-r**) or four (**a**) independent experiments. *P* values are determined by unpaired two-sided t-test in **a-d**, **f**, **g**, **i-k**, **m**, **o**, **q** and **r**. Source data are provided as a Source Data file.

Supplementary Figure4

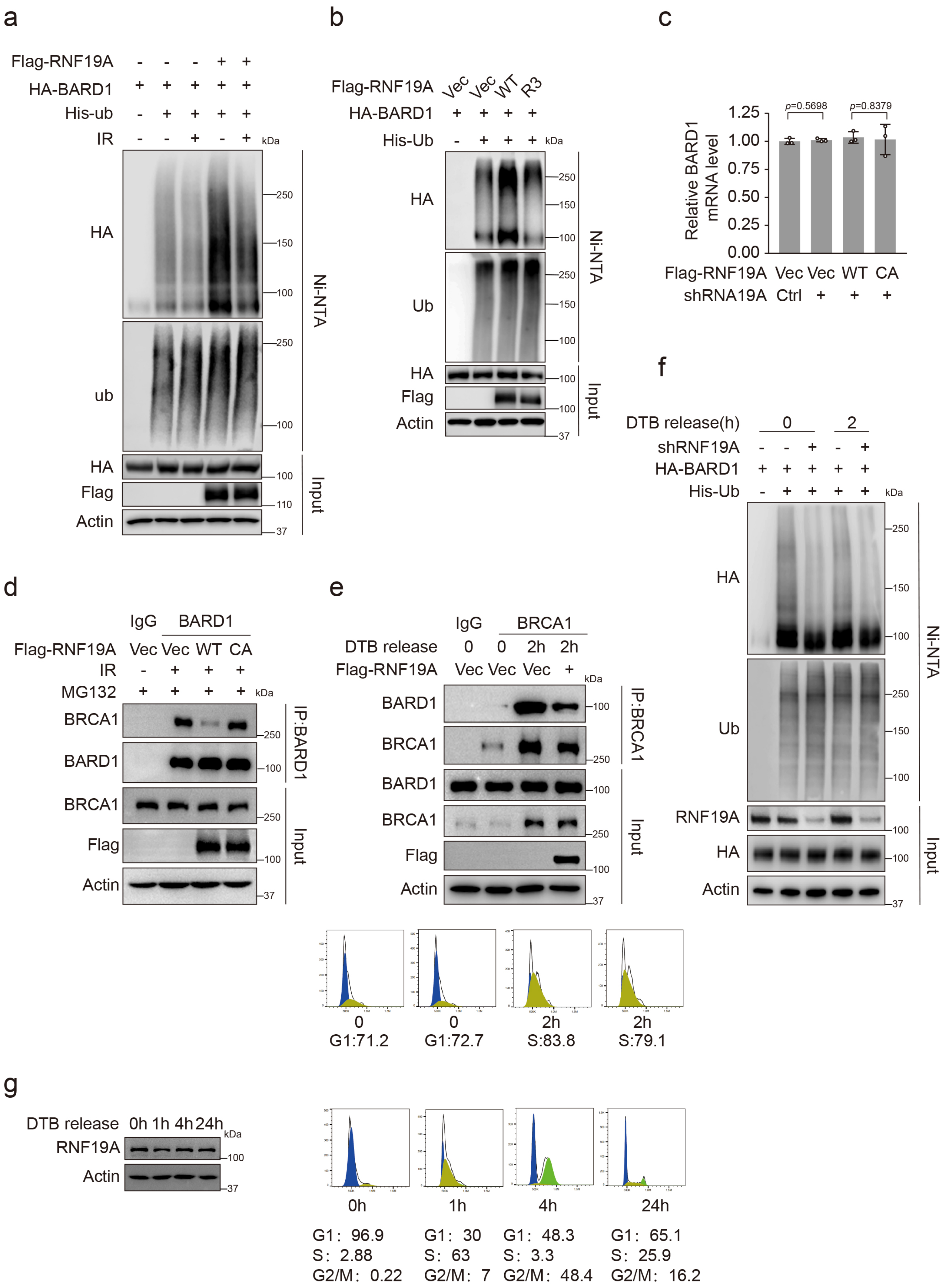

Supplementary Fig.4 RNF19A ubiquitinates BARD1 and is important for BRCA1/BARD1 interaction. a-b HEK293T cells were transfected with indicated plasmids. Cell lysates were subjected to immunoprecipitation with His beads and immunoblotted with the indicated antibodies. **c** mRNA was extracted from control (Ctrl) and RNF19A knockdown MDA-MB-231 cells stably expressing Vec, WT and C316A (CA) Flag-RNF19A, and were subjected to quantitative PCR (qPCR) to detect BARD1 mRNA level. Error bars represent means ± s.d. of three independent experiments and *p* values are determined by unpaired two-sided t-test. **d** HEK293T cells were transfected with Vec, WT or C316A (CA) Flag-RNF19A for 48h and treated with or without IR (10 Gy, 1 h). After 6 h MG132 treating, cell lysates were subjected to immunoprecipitation with control IgG or BARD1 antibodies and immunoblotted with the indicated antibodies. **e** HEK293T cells transfected with Vec or Flag-RNF19A were synchronized in G1 phase by double thymidine block or released into S phase at indicated time points. Cell lysates were subjected to immunoprecipitation with control IgG or BRCA1 antibodies and immunoblotted with the indicated antibodies. The cell cycle profiles were analyzed by flow cytometry. **f** Control or RNF19A knockdown HEK293T cells were transfected with HA-BARD1 and His-Ub for 24 h, and then synchronized by double thymidine block or released into S phase at indicated time points. Cell lysates were subjected to immunoprecipitation with His beads and immunoblotted with the indicated antibodies. **g** U2OS cells were synchronized in G1 phase by double thymidine block or released into indicated time points. Cells were lysed and immunoblotted with the indicated antibodies. Source data are provided as a Source Data file.

Supplementary Figure5

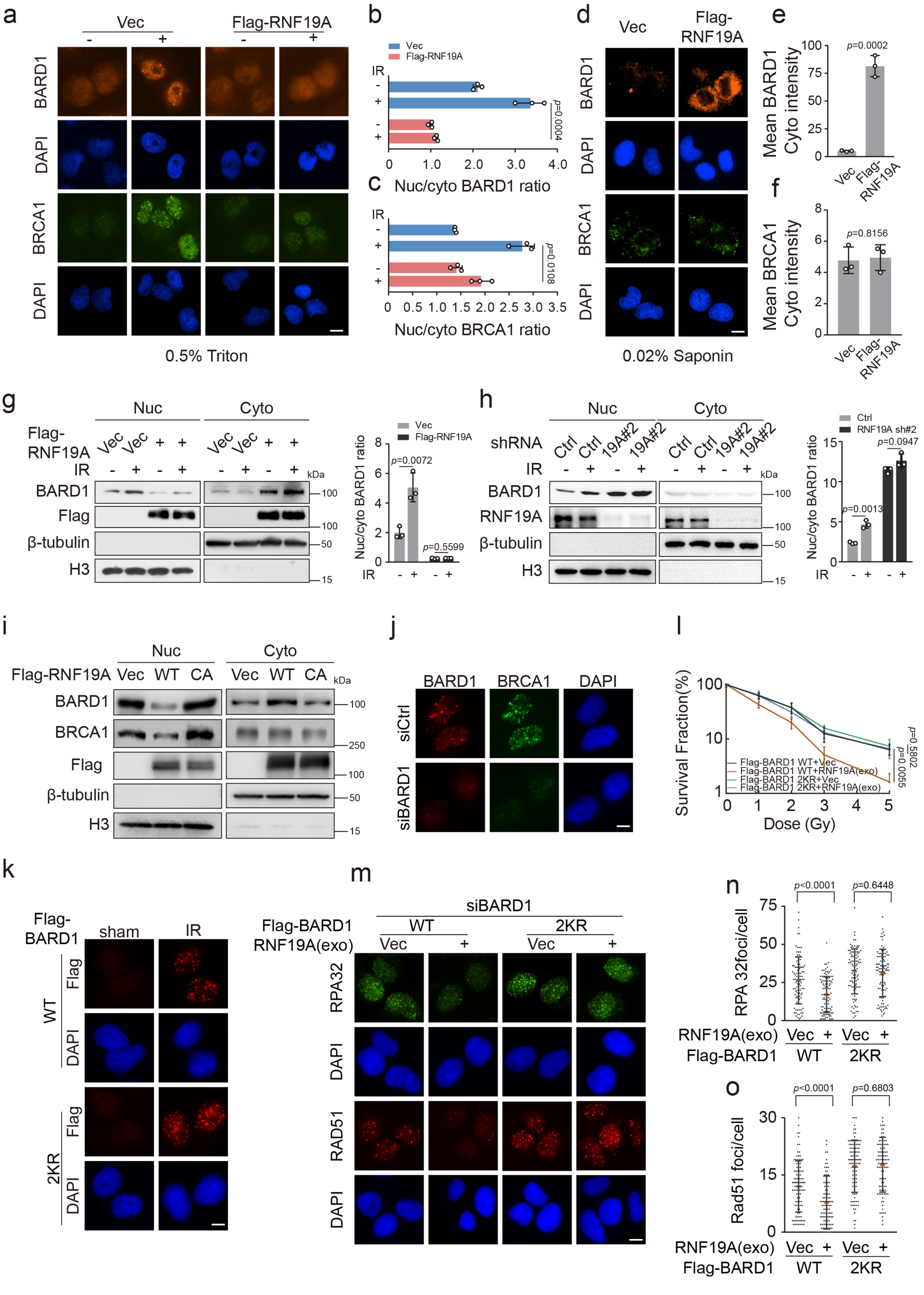

Supplementary Fig. 5 RNF19A's ubiquitination on BARD1 promotes nuclear-to-cytoplasm shuttling of BARD1 and suppresses BRCA1/BARD1 mediated HR. a-c U2OS cells stably expressing Vec or Flag-RNF19A were treated with or without IR (4 Gy, 1 h), fixed with 4% PFA following permeabilized with 0.5% triton. BARD1 and BRCA1 signals were detected by immunofluorescence (**a**). Nuclear (Nuc) and cytoplasmic (Cyto) signals of BARD1 and BRCA1 were quantified and the ratio was calculated by Image J (**b** and **c**). Scale bars, 10μM. **d-f** U2OS shown in (**a**) were treated with IR (4 Gy, 1 h), fixed with 4% PFA following permeabilized with 0.02% saponin. BARD1 and BRCA1 signals were detected by immunofluorescence(**d**). The intensity of cytoplasmic signals of BARD1 and BRCA1 was calculated by Image J (**e** and **f**). Scale bars, 10μM. **g-h** U2OS cells stably expressing Vec or Flag-RNF19A (**g**)/control or RNF19A shRNA (**h**) were treated with or without IR (10 Gy, 1 h). Nuc and Cyto proteins were extracted respectively and immunoblotted with indicated antibodies. Nuc and cyto intensity of BARD1 was quantified by Image J and the ratio was calculated. **i** Nuc and Cyto proteins were extracted respectively from U2OS cells stably expressing Vec, WT and CA Flag-RNF19A. Cell lysates were immunoblotted with indicated antibodies. **j** U2OS cells were transfected with control (Ctrl) or BARD1 siRNAs for 48 h and then treated with IR (2 Gy, 1 h). BARD1 and BRCA1 foci were detected by immunofluorescence. Scale bars, 10μM. **k** U2OS cells stably expressing WT or 2KR Flag-BARD1 were treated with or without IR (2 Gy, 1 h). Flag antibody was used to detect foci signal. Scale bars, 10μM. **l** Sensitivity of indicated cells to IR was assessed by colony formation assay. **m-o** RPA32 and RAD51 foci in indicated cell groups were detected by immunofluorescence (**m**). Quantification of focus signals per cell is shown in (**n**): RPA32 (n=102) and (**o**): RAD51 (n=109). Scale bars, 10μM. Error bars represent means ± s.d. of three independent experiments and p values are determined by unpaired two-sided t-test in **b**, **c**, **e-h**, **l**, **n** and **o**. Source data are provided as a Source Data file.

Supplementary Figure 6

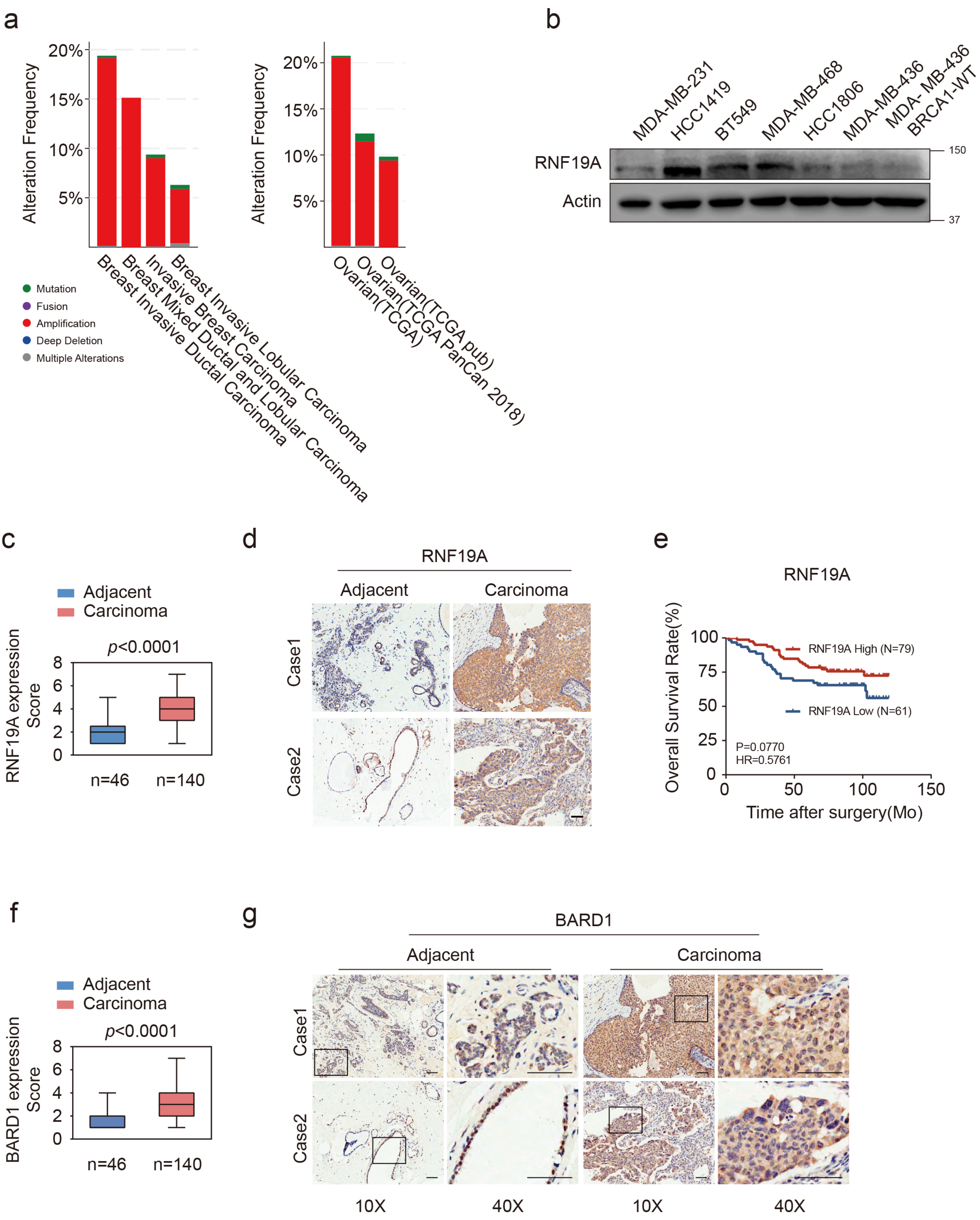

Supplementary Fig.6 The clinical relevance of RNF19A-BARD1 pathway in breast cancer. a Genetic change of RNF19A across different breast and ovarian cancer studies analyzed through cBioportal dataset. **b** Expression of RNF19A in human breast cancer cell lines. **c** IHC staining of RNF19A was evaluated by the German semi-quantitative scoring system dependent on the staining intensity. Adjacent: mini-1, max-5, centre-2, lower quartile-1, upper quartile-2.5; Carcinoma: mini-1, max-7, centre-4, lower quartile-3, upper quartile-5. **d** Representative images of IHC analysis of RNF19A in the serial sections of tumor and paired adjacent normal tissues. Scale bars, 100μM. **e** Kaplan-Meier estimates of overall survival of breast cancer patients with high and low RNF19A expression in a TMA. Log-rank test was used to compare the survival curves between groups. **f** IHC staining of BARD1 was assessed same as RNF19A in (**c**). Adjacent: mini-1, max-4, centre-1, lower quartile-1, upper quartile-2; Carcinoma: mini-1, max-7, centre-3, lower quartile-2, upper quartile-4. **g** Representative images of IHC staining of BARD1 in tumor and paired adjacent normal tissues from two breast cancer patients. Scale bars, 100μM. Source data are provided as a Source Data file.

Supplementary Figure 7

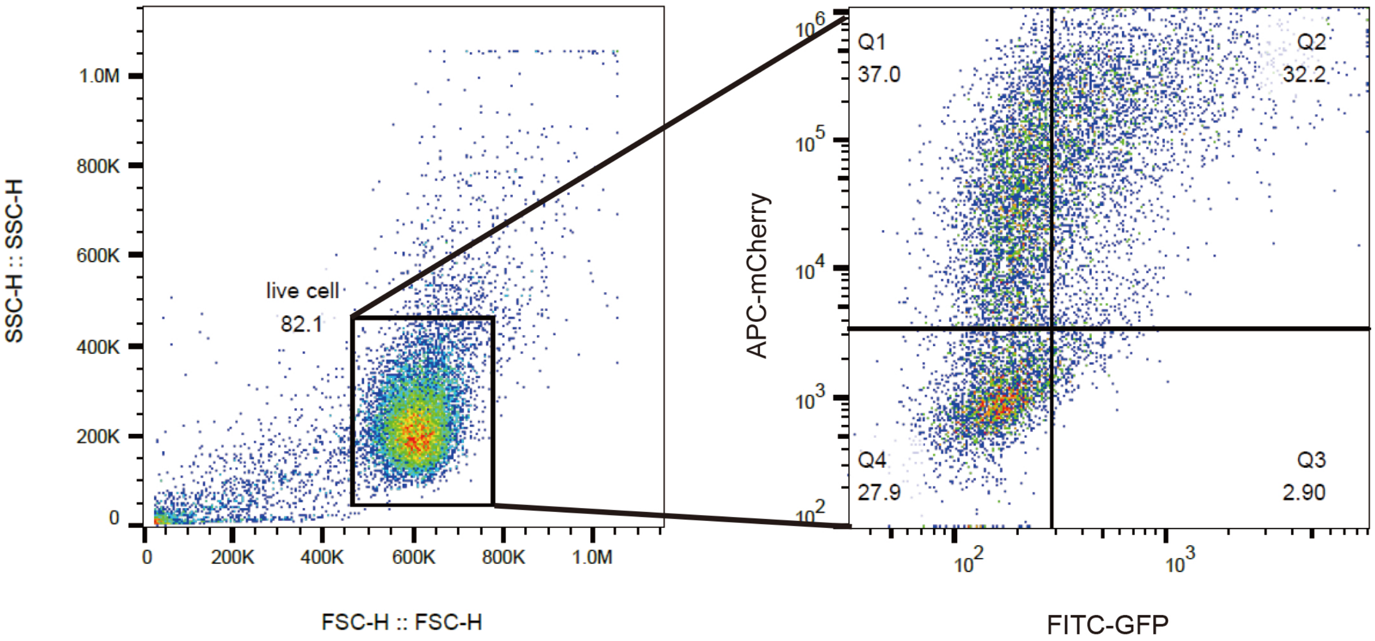

Supplementary Fig. 7 FACS gating strategy for HR and SSA assay. HR/SSA efficiency =Q2/(Q1+Q2)

Supplementary Table1:Correlation between clinical feature and BARD1/RNF19A expression in BC

Clinical character	Clinical groups	All cases	BARD1		X^2	*p* value	RNF19A		X^2	*p* value
			Low	High			Low	High		
Age (years)	≤ 60	81	44	37	1.000	0.317	38	43	0.873	0.350
	> 60	59	27	32			23	36		
Histological grade	I-II	65	40	25	5.205	0.035	27	38	0.032	0.859
	III	65	27	38			28	37		
Tumor size (cm)	< 3	56	33	23	2.326	0.127	24	32	0.034	0.854
	≥ 3	81	37	44			36	45		
Lymphatic metastasis	No	71	37	34	0.113	0.737	25	46	4.095	0.060
	Yes	69	34	35			36	33		
Clinical stage	I-II	98	51	47	0.123	0.726	33	65	14.329	0.0002
	III-IV	39	19	20			27	12		

p values are determined by Pearson's x2 test

Supplementary Table 2. Sequence of oligo nucleotide

**Oligo Name**	**Sequence**
RNF19A NotI For	5'ATAAGAATGCGGCCGCAATGCAAGAACAAGAAATAGGTTTTATCTCTAA-3'
RNF19A XhoI Rev	5'-CCGCTCGAGCTAAATTTCAGTCTGAATTGCAACTTTTAA-3'
RNF19A C316A For	5'-GATGGGAGCAGCAATCACATG-3'
RNF19A C316A Rev	5'-CATGTGATTGCTGCTCCCATC-3'
RNF19A R1(Δ132-179) For	5'-GGAGACTTCATAGAGACTGAACGGTTTAATCCCC-3'
RNF19A R1 (Δ132-179) Rev	5'-GGGGATTAAACCGTTCAGTCTCTATGAAGTCTCC-3'
RNF19A R2(Δ199-264) For	5'-GATGTCTTGATGGATGCTGCTCGACAAG-3''
RNF19A R2 (Δ199-264) Rev	5'-CTTGTCGAGCAGCATCCATCAAGACATC-3'
RNF19A R3 (Δ301-332) For	5'-GATATAAAGCCAATGAAAGAAATCTCAG-3'
RNF19A R3 (Δ301-332) Rev	5'-CTGAGATTTCTTTCATTGGCTTTATATC-3'
BARD1 NotI For	5'-ATAAGAATGCGGCCGCACCGGATAATCGGCAGCCGAGG-3'
BARD1 XhoI Rev	5'-CCGCTCGAGTCAGCTGTCAAGAGGAAGCAAC-3'
BARD1 B1 (Δ1-138) NotI For	5'ATAAGAATGCGGCCGCAAAGAAGAATTCAATTAAAATGTGG-3'
BARD1 ΔNES (Δ92-120) For	5'-CCCCGGCCTGGTCAGATTTGAAAG-3'
BARD1 ΔNES (Δ92-120) Rev	5'- CTTTCAAATCTGACCAGGCCGGGG -3'
BARD1 K96R For	5'-CAAGACTTGCGGATAAATAGACAAC-3'
BARD1 K96R Rev	5'-GTTGTCTATTTATCCGCAAGTCTTG-3'
BARD1 K110R For	5'-CTTTGTAGTCGGCTTCGAAATTTGC-3'
BARD1 K110R Rev	5'-GCAAATTTCGAAGCCGACTACAAAG-3'
