# Supplementary material for: RNF19A-mediated ubiquitination of BARD1 prevents BRCA1/BARD1-dependent homologous recombination
Source: Nat Commun. 2021 Nov 17;12:6653. doi: 10.1038/s41467-021-27048-3 (PMC8599684; doi:10.1038/s41467-021-27048-3)
Supplement: Supplementary file 3 — Reporting Summary [file 41467_2021_27048_MOESM3_ESM.pdf]

nature research

Corresponding author(s): Zhenkun Lou, Jiayi Chen

Last updated by author(s): Oct 27, 2021

Reporting Summary

Nature Research wishes to improve the reproducibility of the work that we publish. This form provides structure for consistency and transparency in reporting. For further information on Nature Research policies, see Authors & Referees and the Editorial Policy Checklist .

Statistics

For all statistical analyses, confirm that the following items are present in the figure legend, table legend, main text, or Methods section.

n/a	Confirmed	
☐	☒	The exact sample size (*n*) for each experimental group/condition, given as a discrete number and unit of measurement
☐	☒	A statement on whether measurements were taken from distinct samples or whether the same sample was measured repeatedly
☐	☒	The statistical test(s) used AND whether they are one- or two-sided<br>*Only common tests should be described solely by name; describe more complex techniques in the Methods section.*
☐	☒	A description of all covariates tested
☐	☒	A description of any assumptions or corrections, such as tests of normality and adjustment for multiple comparisons
☐	☒	A full description of the statistical parameters including central tendency (e.g. means) or other basic estimates (e.g. regression coefficient) AND variation (e.g. standard deviation) or associated estimates of uncertainty (e.g. confidence intervals)
☐	☒	For null hypothesis testing, the test statistic (e.g. *F*, *t*, *r*) with confidence intervals, effect sizes, degrees of freedom and *P* value noted<br>*Give P values as exact values whenever suitable.*
☒	☐	For Bayesian analysis, information on the choice of priors and Markov chain Monte Carlo settings
☒	☐	For hierarchical and complex designs, identification of the appropriate level for tests and full reporting of outcomes
☒	☐	Estimates of effect sizes (e.g. Cohen's *d*, Pearson's *r*), indicating how they were calculated

Our web collection on statistics for biologists contains articles on many of the points above.

Software and code

Policy information about availability of computer code

Data collection	Attune NxT v2.6
Data analysis	GraphPad Prism7, Microsoft Excel 2016 ,flowjo V10, Image J

For manuscripts utilizing custom algorithms or software that are central to the research but not yet described in published literature, software must be made available to editors/reviewers. We strongly encourage code deposition in a community repository (e.g. GitHub). See the Nature Research guidelines for submitting code & software for further information.

Data

Policy information about availability of data

All manuscripts must include a data availability statement. This statement should provide the following information, where applicable:

- Accession codes, unique identifiers, or web links for publicly available datasets
- A list of figures that have associated raw data
- A description of any restrictions on data availability

All the data are available from the corresponding authors upon reasonable request. Source data for figures are provided with this paper.

Field-specific reporting

Please select the one below that is the best fit for your research. If you are not sure, read the appropriate sections before making your selection.

☒ Life sciences ☐ Behavioural & social sciences ☐ Ecological, evolutionary & environmental sciences

For a reference copy of the document with all sections, see nature.com/documents/nr-reporting-summary-flat.pdf

October 2018

Life sciences study design

All studies must disclose on these points even when the disclosure is negative.

Sample size	No statistical method was used to predetermine sample size. Required sample sizes were estimated based on our experience performing similar experiments in previous publications. Sample sizes are illustrated in figure legends.
Data exclusions	No samples were excluded from analysis.
Replication	All results were tested and confirmed with at least three independent experiments.
Randomization	All samples such as cells or animals were randomly allocated into experimental groups.
Blinding	No blinding assessment was performed as microscopy and FACS data. For manual microscopy, Data were collected randomly choose at least 5 region. For FACs and high-content microscopy data were anlayzed automatically. One individual performed the experiment while another individual (blinded to the group allocation) performed the analysis.For colony-forming units quantification, data were collected blindly by technicians without information of the research purpose.For immunoblotting, immunoprecipitation and qPCR experiments, samples of different groups were fairly collected and generated simultaneously, and all data are obtained and presented in an unbiased manner.

Reporting for specific materials, systems and methods

We require information from authors about some types of materials, experimental systems and methods used in many studies. Here, indicate whether each material, system or method listed is relevant to your study. If you are not sure if a list item applies to your research, read the appropriate section before selecting a response.

Materials & experimental systems

n/a	Involved in the study	
☐	☒	Antibodies
☐	☒	Eukaryotic cell lines
☒	☐	Palaeontology
☐	☒	Animals and other organisms
☐	☒	Human research participants
☒	☐	Clinical data

Methods

n/a	Involved in the study	
☒	☐	ChIP-seq
☐	☒	Flow cytometry
☒	☐	MRI-based neuroimaging

Antibodies

Antibodies used

The following antibodies were used:
Anti-RNF19A (Abcam,ab251750, WB-1:500, IHC-1:200, rabbit)
Anti-BARD1 (Genetex,GTX132094, WB-1:1000, IF-1:1000, IHC-1:500,rabbit)
Anti-RAD51 (Genetex,GTX100469, WB-1:1000, IF-1:1000,rabbit)
Anti-BRCA1 (Santa cruz Biotechnology,sc-6954 WB-1:1000, IF-1:1000,mouse)
Anti-Ub (Santa cruz Biotechnology,sc-8017, WB-1:1000,mouse)
Anti-GFP (Santa cruz Biotechnology,sc-9996, WB-1:1000,mouse)
Anti-CtIP (Santa cruz Biotechnology,sc-271339 WB-1:1000,mouse)
Anti-RPA3232 (Santa cruz Biotechnology,sc-56770, IF-1:1000,mouse)
Anti-γ-H2AX (Sigma-Aldrich,05-636, IF-1:1000,mouse)
Anti-FK2 (Sigma-Aldrich,04-263, IF-1:1000,mouse)
Anti-MDC1 (Sigma-Aldrich,05-1572, IF-1:1000,mouse)
Anti-CtIP (Active Motif,61141 IF-1:1000)
Anti-53BP1 (Novus Biologicals, NB100-304, WB-1:1000, IF-1:1000,Rabbit)
Anti-FLAG (F1804,mouse and F7425 rabbit, WB-1:3000, IF-1:1000)
Anti-HA (Sigma-Aldrich,H9658, WB-1:3000,mouse)
Anti-β-actin (Sigma-Aldrich,A2228, WB-1:3000,mouse)
Normal rabbit IgG (12-370) and mouse IgG (12-371) were purchased from Sigma-Aldrich.
Alexa Fluor® 488 AffiniPure Donkey Anti-Rabbit IgG (H+L, 715-585-150), Alexa Fluor® 594 AffiniPure Donkey Anti-Rabbit IgG (H+L, 711-585-152), Alexa Fluor® 488 AffiniPure Donkey Anti-Mouse IgG (H+L ,715-545-150), and Alexa Fluor® 594 AffiniPure Donkey Anti-Mouse IgG (H+L, 715-585-150) were purchased from Jackson ImmunoResearch.
Donkey Anti-Mouse IgG (H+L, 715-675-151) and Donkey Anti-Rabbit IgG (H+L, 711-675-152) were purchased from Jackson ImmunoResearch.

Validation

Anti-RNF19A (Abcam, ab251750, rabbit). Reacts with: Mouse, Human, Pig. Suitable for: IHC, WB. Citation from manufacturer is listed at https://www.abcam.com/dorfin-antibody-ab251750.html.

Anti-BARD1 (Genetex, GTX132094, rabbit). Reacts with: Mouse, Human, Rat. Suitable for: WB, IP, IF. Citation from manufacturer is listed at https://www.genetex.cn/Product/Detail/BARD1-antibody/GTX132094.
Anti-RAD51 (Genetex ,GTX100469, rabbit). Reacts with: Mouse, Human, Rat, Zebrafish. Suitable for: WB, IP, IF. Citation from manufacturer is listed at https://www.genetex.cn/Product/Detail/Rad51-antibody-N1C2/GTX100469.
Anti-BRCA1 (Santa cruz Biotechnology, sc-6954, mouse). Reacts with: Mouse, Human, Rat, etc. Suitable for: WB, IF, IP, ELISA. Citation from manufacturer is listed at https://www.scbt.com/p/brca1-antibody-d-9?productCanUrl=brca1-antibody-d-9&_requestid=2191735.
Anti-Ub (Santa cruz Biotechnology, sc-8017, mouse). Reacts with: mouse, rat, human and Drosophila. Suitable for: WB, IF, IP, ELISA. Citation from manufacturer is listed at WB, IF, IP,IHC, etc. Citation from manufacturer is listed at https://www.scbt.com/p/ubiquitin-antibody-p4d1?requestFrom=search.
Anti-GFP (Santa cruz Biotechnology, sc-9996, mouse). Reacts with: mouse, rat, human,etc. Suitable for: WB, IF, IP, ELISA, etc. Citation from manufacturer is listed at https://www.scbt.com/p/gfp-antibody-b-2?requestFrom=search.
Anti-CtIP (Santa cruz Biotechnology, sc-271339, mouse). Reacts with: mouse, rat, human. Suitable for: WB, IF, IP, ELISA. Citation from manufacturer is listed at https://www.scbt.com/p/ctip-antibody-d-4?requestFrom=search.
Anti-RPA3232 (Santa cruz Biotechnology, sc-56770, mouse). Reacts with: mouse, rat, human. Suitable for: WB, IF, IP, ELISA, etc. Citation from manufacturer is listed at https://www.scbt.com/p/rpa-32-kda-subunit-antibody-9h8?requestFrom=search.
Anti-γ-H2AX (Sigma-Aldrich,05-636, mouse). Reacts with: Vertebrates. Suitable for: WB, IF, IHC, ChIP. Citation from manufacturer is listed at https://www.sigmaaldrich.cn/CN/en/product/mm/05636?context=product.
Anti-FK2 (Sigma-Aldrich,04-263, mouse). Reacts with: All. Suitable for: ELISA, WB, IP, IF. Citation from manufacturer is listed at https://www.merckmillipore.com/CN/en/product/Anti-Ubiquitinylated-proteins-Antibody-clone-FK2,MM_NF-04-263?ReferrerURL=https%3A%2F%2Fcn.bing.com%2F.
Anti-MDC1 (05-1572, IF-1:1000, mouse). Reacts with: Mouse, Human, Chimpanzee, Bovine. Suitable for: ICC, WB. Citation from manufacturer is listed at https://www.merckmillipore.com/CN/zh/product/Anti-MDC1-Antibody-clone-P2B11,MM_NF-05-1572.
Anti-CtIP (Active Motif ,61141, mouse). Reacts with: Human. Suitable for: IP, IF, ChIP, WB. Citation from manufacturer is listed at https://www.activemotif.com/catalog/details/61141/ctip-antibody-mab-clone-14-1.
Anti-53BP1 (Novus Biologicals, NB100-304, rabbit). Reacts with: Mouse, Human, Rat, etc. Suitable for: WB, IP, IF, etc. Citation from manufacturer is listed at https://www.novusbio.com/products/53bp1-antibody_nb100-304
Anti-FLAG (Sigma-Aldrich, F1804, mouse). Reacts with: All. Suitable for: WB, IP, IF, etc. Citation from manufacturer is listed at https://www.sigmaaldrich.cn/CN/en/product/sigma/f1804?context=product.
Anti-FLAG (Sigma-Aldrich, F7425, rabbit). Reacts with: All. Suitable for: WB, IP, IF, etc. Citation from manufacturer is listed at https://www.sigmaaldrich.cn/CN/en/product/sigma/f7425?context=product.
Anti-HA (Sigma-Aldrich, H9658, mouse). Reacts with: All. Suitable for: WB, IP, ELISA, ICC. Citation from manufacturer is listed at https://www.sigmaaldrich.cn/CN/en/product/sigma/h9658?context=product.
Anti-β-actin (Sigma-Aldrich, A2228, mouse). Reacts with: All. Suitable for: WB, IHC, ARR, ICC. Citation from manufacturer is listed at https://www.sigmaaldrich.cn/CN/en/product/sigma/a2228?context=product.
Normal rabbit IgG (Sigma-Aldrich, 12-370) Suitable for: WB, IP. Citation from manufacturer is listed at https://www.merckmillipore.com/CN/zh/product/Normal-Rabbit-IgG,MM_NF-12-370?ReferrerURL=https%3A%2F%2Fcn.bing.com%2F
Normal mouse IgG (Sigma-Aldrich, 12-371) Suitable for: WB, IP. Citation from manufacturer is listed at https://www.merckmillipore.com/CN/zh/product/Normal-Mouse-IgG,MM_NF-12-371
The commercial secondary antibodies were validated by manufactures.

Eukaryotic cell lines

Policy information about cell lines

Cell line source(s)	HEK293T (ATCC)<br>U2OS (ATCC)<br>MDA-MB-231(ATCC)<br>HCC1806(ATCC)<br>ER-AsiSI U2OS cells [generated by Dr. Gaëlle Legube's lab (University of Toulouse, France)]<br>Clz3 cells [provided by Dr. Zhiyong Mao (Tongji University, China)]
Authentication	The Cell lines have been authenticated based on morphological criteria.
Mycoplasma contamination	All cell lines were tested negative for mycoplasma.
Commonly misidentified lines<br>(See ICLAC register)	No commonly misidentified cell lines were used.

Animals and other organisms

Policy information about studies involving animals; ARRIVE guidelines recommended for reporting animal research

Laboratory animals	Outbred athymic nude mice. 5- week-old female mice are used in experiments. Mice were housed in groups (3–5 per cage) at 22–24 °C with a 12 h light-dark cycle and ad libitum access to regular chow diet and water.
Wild animals	No wild animals were used in this study.
Field-collected samples	No filed-collected samples were used in this study.

Ethics oversight	All animal work was approved by the Institutional Animal Care and Use Committee (IACUC) at Mayo Clinic (Rochester,MN) under protocol A00002864-17

Note that full information on the approval of the study protocol must also be provided in the manuscript.

Human research participants

Policy information about studies involving human research participants

Population characteristics	Clinical human breast cancer used in microarray analysis were histopathologically and clinically diagnosed. Among the cohort of 140 female breast cancer patients, there are 46 pairs of breast cancer and adjacent tissues plus 94 breast cancer tissue samples. The details of patients were provided in Supplementary Table 1.
Recruitment	Tissue microarray of human breast tumors and paired adjacent normal tissues were purchased from Shanghai OUTDO Biotech Co, Ltd (Shanghai). The selection of patients was random and we believe that there was no bias in data selection.
Ethics oversight	All experiments from human samples were performed with the approval of the Medical Ethic Committee of Shanghai JiaoTong University School of Medicine. Informed consent was obtained from all individuals.

Note that full information on the approval of the study protocol must also be provided in the manuscript.

Flow Cytometry

Plots

Confirm that:

- [x] The axis labels state the marker and fluorochrome used (e.g. CD4-FITC).
- [x] The axis scales are clearly visible. Include numbers along axes only for bottom left plot of group (a 'group' is an analysis of identical markers).
- [x] All plots are contour plots with outliers or pseudocolor plots.
- [x] A numerical value for number of cells or percentage (with statistics) is provided.

Methodology

Sample preparation	We used FACS for cell cycle analysis (cell cycle and sub G1) or HR/NHEJ analysis. Cells were transfected with m-cherry/GFP expression plasmids or PI , trypsinized and suspended in PBS. Further details of the experimental procedures are provided in the materials and Methods.
Instrument	Attune NxT Flow Cytometer (Thermo fisher SCIENTIFIC)
Software	Attune NxT Flow Cytometer software v2.6
Cell population abundance	No flow based sorting was performed.
Gating strategy	The FSC/SSC gates defined the single cell population and gated indicated antibodies positive population. For cell cycle, after FSC/SSC gating, PI-A and PI-H were gated for single cell population and analyzed PI-A positive population as cell cycle distribution.

- [x] Tick this box to confirm that a figure exemplifying the gating strategy is provided in the Supplementary Information.
